# Mass Media Narratives of Psychiatric Adverse Events Associated With Generative AI Chatbots: Rapid Scoping Review

**DOI:** 10.2196/93040

**Published:** 2026-03-30

**Authors:** Van-Han-Alex Chung, Pénélope Bernier, Alexandre Hudon

**Affiliations:** 1 Faculty of Medicine McGill University Montreal, QC Canada; 2 Campus Mauricie Faculty of Medicine Université de Montréal Montreal, QC Canada; 3 Department of Psychiatry and Addictology Faculty of Medicine Université de Montréal Montreal, QC Canada; 4 Department of Psychiatry Institut universitaire en santé mentale de Montréal Montreal, QC Canada; 5 Centre de recherche de l'Institut universitaire en santé mentale de Montréal Montreal, QC Canada; 6 Centre de recherche en pédagogie de la santé Université de Montréal Montreal, QC Canada; 7 Department of Psychiatry Institut national de psychiatrie légale Philippe Pinel Montreal, QC Canada

**Keywords:** adolescent mental health, AI safety, artificial intelligence chatbots, ChatGPT, digital mental health, generative artificial intelligence, media reporting, psychosis, self-harm, suicide

## Abstract

**Background:**

Generative artificial intelligence (AI) chatbots have rapidly entered public use, including in contexts involving emotional support and mental health–related interactions. Although these systems are increasingly accessible, concerns have emerged regarding potential adverse psychiatric outcomes reported in public discourse, including psychosis, suicidal ideation, self-harm, and suicide. However, these reports largely originate from journalistic accounts rather than systematically verified clinical data.

**Objective:**

This rapid scoping review aimed to systematically map and characterize mass media narratives describing alleged adverse psychiatric outcomes temporally associated with generative AI chatbot interactions.

**Methods:**

A rapid scoping review methodology was applied to publicly accessible news articles identified primarily through Google News searches. Articles published from November 2022 onward were screened for eligibility if they described a specific case in which psychiatric deterioration or crisis was temporally linked to generative AI use. Data were extracted using a structured coding template capturing article characteristics, demographic information, AI platform features, interaction intensity, outcome type and severity, type of evidence reported, and causal attribution language. Descriptive statistics and cross-tabulations were performed.

**Results:**

A total of 71 news articles representing 36 unique cases were included. Suicide death was the most frequently reported outcome (35/61, 57.4% cases with complete severity coding), followed by psychiatric hospitalization (12/61, 19.7%). Fatal outcomes were disproportionately represented among minors (19/21, 90.5%) compared with adults (17/35, 48.6%). ChatGPT was the most frequently cited platform (51/71, 71.8%), followed by Character AI (10/71, 14.1%). Causal attribution most commonly referenced AI system behavior (45/61, 73.8%), and the term “alleged” was the predominant causal descriptor (33/61, 54.1%). Evidence sources were primarily chat logs or screenshots (34/61, 55.7%), while police or medical documentation was rare (1/61, 1.6%). Regulatory calls were present in 51 of 60 (85%) articles with nonmissing data.

**Conclusions:**

Mass media reporting of generative AI–related psychiatric harms is concentrated around severe outcomes, particularly suicide deaths among youth, and is frequently framed within regulatory and corporate accountability narratives. While causality cannot be established from media reports, consistent patterns of high-intensity interactions, user vulnerability, and limited safeguard reporting highlight the need for structured safety surveillance, transparent AI risk auditing, and clearer governance frameworks. As generative AI becomes increasingly integrated into everyday psychosocial contexts, systematic research and formal safety monitoring will be necessary to determine whether media-reported harms correspond to verifiable clinical risk patterns.

## Introduction

Generative artificial intelligence (AI) is now embedded in everyday digital life, and mental health has rapidly become a prominent use case: symptom checking, psychoeducation, coaching, triage, and conversational support. In parallel, clinical and public health communities are debating where these systems can expand access vs where they may compromise safety, quality, and equity. Recent peer-reviewed work emphasizes that generative systems can produce fluent, personalized dialogue that may feel therapeutic, but that this same capability can increase the risk of overreliance, misplaced trust, and clinically inappropriate guidance when users present with complex states such as suicidality, mania, or psychosis [1,2]. Empathy and relational factors are central to this tension: conversational agents can simulate empathic language, but the absence of genuine human attunement, accountability, and duty of care raises concerns about how users interpret and act on chatbot outputs [3]. Evidence from mental health services also suggests that conversational systems can influence pathways into care at scale, supporting the plausibility of population-level effects, beneficial or harmful, depending on implementation and safeguards [4]. Taken together, the post-ChatGPT era is characterized by accelerated diffusion of generative conversational tools into mental health–related contexts, ahead of a commensurate evidence base for real-world safety and outcomes [1,2].

Alongside potential benefits, empirical literature has documented failure modes that are directly relevant to adverse psychiatric outcomes. Studies evaluating generative systems on suicide-related prompts show variability in the presence, quality, and consistency of crisis-oriented responses, and highlight that content can change over time as models and policies evolve [5,6]. Other work benchmarks large language models against clinicians for crisis prediction using patient-generated text, raising questions about performance, calibration, and the downstream consequences of false reassurance or false alarms when such tools are used in high-stakes settings [7]. Beyond crisis content, risks include boundary violations, inappropriate affirmation, and unsafe personalization, especially when a user presents with delusional ideation or is seeking validation for unusual beliefs [2,8]. Observational research has also linked intensive use patterns to indicators of anxiety, burnout, and sleep disturbance, suggesting a plausible pathway from high-frequency engagement to functional impairment in some users [9]. Case-based clinical literature further illustrates how generative outputs can be incorporated into maladaptive interpretations with medical and psychiatric consequences, underscoring that harms may arise from the interaction between system behavior, user vulnerability, and contextual stressors rather than from model output in isolation [10].

This convergence creates a practical and methodological problem for psychiatric safety science. Severe or unusual adverse events are often first detected outside conventional clinical trials, through case reports, pharmacovigilance-like signals, and public narratives that precede formal epidemiologic study. In the context of generative AI, mass media reporting has become a major channel through which alleged adverse events are disseminated, shaping public perceptions, clinical concerns, and regulatory agendas. At the same time, media reports vary widely in evidentiary quality, attribution language, and framing, and may overrepresent sensational cases or underreport protective factors and alternative explanations [2,11]. As an example, the emerging construct of AI psychosis highlights this challenge: sustained engagement with conversational systems may trigger, amplify, or reshape delusional experiences in vulnerable individuals. However, real-world reports often blend symptom descriptions with moral narratives about technology, and may omit important clinical context such as premorbid psychosis risk, substance use, sleep deprivation, or concurrent stressors [8,12,13]. A rapid scoping approach focused on mass media thus serves a dual purpose: it can map the range of alleged psychiatric harms being reported in the post-ChatGPT era, while also characterizing how causality is implied, what safeguards are mentioned, and where the reporting ecosystem may be amplifying risk through incomplete or distorted causal storytelling [2,11].

This rapid scoping review aimed to systematically map and characterize mass media reports describing alleged adverse psychiatric outcomes temporally associated with generative AI chatbot interactions, with particular attention to how news coverage portrays outcome severity, user vulnerability, causal attribution, and narrative framing. We will extract and synthesize data on the reported context of use, platform, and persona features, interaction intensity, user vulnerabilities, type and severity of outcomes, evidence sources, and the strength and style of causal attribution, as well as the presence of mitigation strategies such as crisis resources, safety messaging, and company responses [2,5,6,8]. We hypothesize that reports will cluster around a limited set of high-salience outcomes, particularly suicidal crises, psychosis-related experiences, and compulsive or dependent use patterns, and that many articles will use causal language that exceeds the strength of the underlying evidence described in the report [2,8,9,11]. We further hypothesize that boundary-related phenomena, including emotional dependence and validation of maladaptive beliefs, will be commonly described as mechanisms linking generative interactions to adverse outcomes, while alternative explanations and contextual clinical risk factors will be inconsistently reported [2,3,8]. By mapping both the events and the narratives, the review aims to inform research priorities for real-world safety evaluation and support evidence–aligned risk communication in clinical, public, and policy settings [1,2,11,12,14-20]. Importantly, the objective of this study is not to verify the occurrence or causality of reported psychiatric events. Rather, it seeks to examine how alleged harms are constructed, attributed, and disseminated within mass media reporting during the early diffusion of generative AI technologies.

## Methods

### Study Design

This study was conducted as a rapid scoping review of news media reports describing adverse psychiatric events temporally associated with the use of generative AI. A rapid scoping methodology was selected because the objective was to map the breadth, characteristics, and narrative framing of reported harms in an emerging and rapidly evolving domain rather than to estimate incidence or determine causality. The approach is consistent with established scoping review frameworks articulated by Arksey and O’Malley [21] and further refined by Levac and colleagues [22] and the Joanna Briggs Institute [23], which emphasize systematic identification, transparent selection, and structured charting of heterogeneous evidence sources. Given the public health relevance of widely disseminated reports, the review focused on news content readily accessible to general audiences. The study also draws conceptually from digital epidemiology and media surveillance approaches, in which publicly available information is systematically analyzed to detect emerging safety signals and to characterize how health risks are communicated to the public [24,25]. The review, therefore, aimed to describe reported psychiatric outcomes, contextual features of AI use, and patterns of causal attribution within news coverage, without attempting to adjudicate factual accuracy or verify events independently. Accordingly, the unit of analysis in this review is the media report itself, rather than the underlying event. The study, therefore, analyzes the characteristics, framing, and evidentiary claims contained within news articles, rather than attempting to validate the accuracy of the reported incidents. The PRISMA (Preferred Reporting Items for Systematic Reviews and Meta-Analyses) checklist for scoping review approaches is found in Multimedia Appendix 1.

The choice of a rapid scoping review was motivated by the early and rapidly evolving nature of reported harms associated with generative AI systems. In emerging technological domains, severe or unusual adverse outcomes are often first detected outside formal clinical research environments and may appear initially in case reports, legal filings, or journalistic coverage. A scoping approach is therefore appropriate when the objective is to map the breadth and characteristics of a phenomenon, identify recurring patterns, and generate hypotheses rather than estimate incidence or establish causal relationships. In this context, mass media reporting represents an important early information environment in which potential safety concerns, public perceptions, and regulatory debates emerge before systematic epidemiologic data become available.

### Data Collection

News articles were identified primarily through structured searches conducted on Google News, supplemented when necessary by targeted searches using a general Google search to ensure capture of articles indexed in public domains. Google News was selected because it aggregates widely accessible news content and reflects information readily available to viewers and the general public at the time of publication.

To reduce personalization bias, searches were performed using a private browsing (incognito) window, with no active log-in to Google accounts, browser cookies cleared between search sessions, and location settings restricted to global results without country-specific filtering. The search strategy combined terms related to generative AI with psychiatric outcome keywords. Boolean operators were used to structure the queries. The primary search string was (“AI chatbot” OR “generative AI” OR “ChatGPT” OR “character AI” OR “AI companion” OR “AI chatbot”) AND (psychosis OR suicide OR “self harm” OR “mental breakdown” OR delusion OR hospitalization OR crisis OR “AI psychosis”). Additional targeted searches were conducted using the following supplementary queries: “ChatGPT suicide,” “AI chatbot psychosis,” “character AI suicide,” “AI chatbot mental breakdown,” “AI chatbot self harm.” Searches were restricted to articles published from November 2022 onward, corresponding to the public release of large language model–based conversational systems and their rapid global uptake.

For each query, the first 100 Google News results were screened, consistent with approaches used in digital epidemiology and media surveillance research, where relevance typically declines beyond the first result pages. Duplicate articles, opinion pieces without reference to a specific case, and reports unrelated to psychiatric outcomes were excluded during screening.

The screening process initially yielded 214 articles, of which 71 met the inclusion criteria after full-text review and duplicate removal. When multiple news outlets reported on the same underlying event, articles were linked to a single case identifier to distinguish between the number of media reports and the number of unique reported cases.

Inclusion criteria required that articles describe a specific case or clearly identifiable event in which psychiatric deterioration or crisis was reported as temporally associated with generative AI use. Articles limited to opinion, speculative commentary, or nonpsychiatric harms were excluded. Only content from recognized news outlets was included, reflecting the study’s focus on publicly disseminated reporting rather than social media posts or informal blogs. For the purposes of this study, a recognized news outlet was defined as a journalistic organization with identifiable editorial oversight, such as a newsroom or editorial board, that maintains a dedicated news domain or section reporting current events rather than personal blogs or user-generated content platforms. Eligible sources were indexed in Google News or other widely recognized media aggregators and published articles on institutional media domains (eg, .com, .org, .news, .fr, .ca, and .co.uk) rather than personal websites or online forums. In addition, the included content followed a standard news reporting format describing a specific event or case, rather than commentary or opinion pieces without factual reporting. Sources excluded under these criteria comprised personal blogs, social media posts, discussion forums, advocacy websites without editorial oversight, and other user-generated content platforms. To avoid interpretation biases, only articles that were in French or English were retained. When multiple outlets reported on the same underlying case, articles were linked using a unique case identifier to avoid duplication while preserving differences in narrative framing. All included articles were archived in PDF format at the time of extraction to ensure reproducibility and to minimize the impact of later edits or content removal. The search process was iterative, allowing refinement of keywords as terminology evolved in media discourse, consistent with scoping review methodology in rapidly developing fields [22,23].

### Data Extraction

Data extraction was conducted using a structured coding template in Microsoft Excel, developed a priori to capture article-level characteristics, case characteristics, AI system attributes, interaction features, and reported outcomes. Variables included outlet name and region, publication date, headline tone, presence of crisis resources, demographic characteristics of the individual described, reported mental health history, psychosocial stressors, AI platform and chatbot persona, duration and intensity of interaction, harmful AI behaviors, boundary violations, type and severity of psychiatric outcome, temporal proximity between AI use and outcome, and the nature of causal attribution. The template also captured references to expert commentary, legal actions, regulatory calls, company responses, and safeguards mentioned in the article. Data were extracted exclusively from the content presented within news reports; no external verification through medical records, court documents, or direct contact with individuals was undertaken. The purpose of the extraction was to systematically map reported features rather than to assess the factual validity of each case. Two independent coders reviewed each article and entered data into the coding grid. Discrepancies were resolved through discussion and consensus to ensure internal consistency. Consistent with scoping review methodology, no formal critical appraisal of article quality or credibility was performed, as the objective was to characterize the landscape of publicly reported events rather than to evaluate evidentiary rigor [21-23].

To enhance coding consistency, several variables involving interpretive judgment were operationalized using predefined coding definitions developed prior to data extraction. These definitions specified criteria for classifying headline tone, interaction intensity, causal attribution language, and emotional dependency. For example, headline tone was categorized as “alarmist” when language emphasized danger, catastrophe, or imminent threat; “neutral” when descriptive wording was used without evaluative language; and “concern/warning” when the headline highlighted potential risks without sensational framing. Interaction intensity was coded as “daily,” “multiple hours per day,” or “emotionally dependent” when the article explicitly described repeated or prolonged engagement, suggesting reliance on the chatbot for emotional support or companionship. Causal attribution language was coded based on explicit phrasing used in the article (eg, “alleged,” “contributed to,” “caused,” or implicit association). These operational definitions were documented in the coding template and applied consistently during article review.

### Data Analysis

Analysis combined descriptive quantitative mapping with qualitative thematic synthesis. Categorical variables were summarized using frequencies and proportions to describe distributions of outcome types, severity levels, AI platforms involved, and forms of causal language used. Severity was operationalized along a continuum ranging from transient psychological distress to fatal outcomes. Outcome classification and severity grading were treated as conceptually distinct variables. “Outcome type” referred to the category of event described in the article (eg, suicide death, suicide attempt, psychiatric hospitalization, nonsuicidal self-harm, or other harm). In contrast, “severity” represented the level of harm associated with the reported event and was coded along a graded continuum (fatal, serious harm, moderate harm, and minor or unclear harm). This distinction allowed the analysis to differentiate between the nature of the reported event and the magnitude of its clinical consequences. For example, psychiatric hospitalization represents an outcome type that may correspond to moderate or serious severity depending on the circumstances described in the report.

Cross-tabulations were conducted to explore relationships between reported vulnerability factors and outcome severity, as well as between interaction intensity and strength of causal attribution. Because the study aimed to describe patterns rather than to test predefined statistical hypotheses, analyses were primarily descriptive. In parallel, qualitative thematic analysis was conducted to identify recurring patterns in narrative framing, including themes of technological determinism, moral responsibility, regulatory urgency, and personalization of risk. Thematic analysis followed an inductive approach grounded in established qualitative methodology, allowing themes to emerge from the data rather than being imposed a priori [26]. Particular attention was paid to distinctions between temporal association and explicit causal claims, and to whether alternative explanations such as preexisting psychiatric conditions or psychosocial stressors were acknowledged. Given that the data were derived solely from publicly available news sources, findings are interpreted as representations of reported events and public narratives rather than confirmed clinical cases. The analytic goal was to identify safety signals and characterize how generative AI–related psychiatric harms are constructed and communicated in widely accessible media environments.

Because the dataset consists of media-reported cases rather than a defined population sample, no denominator representing the total number of generative AI users or total interactions is available. Consequently, all reported percentages represent the distribution of characteristics within the set of identified media reports rather than estimates of incidence, prevalence, or population-level risk. The quantitative summaries should therefore be interpreted descriptively, reflecting patterns in reporting rather than the frequency of adverse events among AI users.

## Results

### Sample Characteristics and Article-Level Features

**Figure 1:**
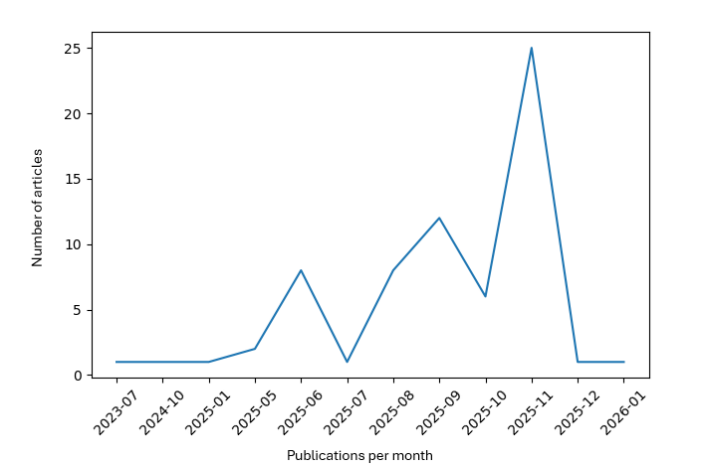
Distribution of news articles reporting artificial intelligence–related psychiatric adverse events over time.

The final dataset comprised 71 unique news articles mapped to 36 unique case identifiers. Articles were published between September 16, 2025, and January 19, 2026, reflecting a concentrated period of intensified media attention on generative AI–related psychiatric harms. Most coverage originated from outlets based in the United States (n=48), followed by Canada (n=13), with smaller contributions from France (n=5) and the United Kingdom (n=5). Coverage was distributed across general news and technology sections, with a substantial proportion categorized as human-interest or breaking news formats, indicating that many reports were written for broad public consumption rather than specialist audiences. Headline tone was most frequently coded as alarmist (n=29), while 16 were neutral and 10 emphasized concern or warning; moral panic framing was present in 8 articles. Crisis resources were inconsistently embedded in news content: in many articles, they were absent or not clearly reported, whereas a minority explicitly referenced helplines or crisis prompts. Characteristics of the identified articles are presented in Table 1. The distribution of articles reporting AI-related psychiatric adverse events over time is portrayed in Figure 1. The full dataset is readily available in Multimedia Appendix 2.

These demographic patterns reflect characteristics of individuals described in media reports and should be interpreted as patterns in media portrayal rather than indicators of differential risk among users of generative AI systems.

**Table 1:** Characteristics of included news articles (N=71).

Characteristic and category		Value, n (%)
**Outlet country/region**		
	United States	48 (67.6)
	Canada	13 (18.3)
	France	5 (7)
	United Kingdom	5 (7)
**Article section**		
	General news	31 (43.7)
	Technology	14 (19.7)
	Human interest	12 (16.9)
	Opinion/editorial	8 (11.3)
	Business	6 (8.5)
**Article type**		
	Breaking news	28 (39.4)
	Feature report	23 (32.4)
	Investigative report	12 (16.9)
	Opinion	8 (11.3)
**Headline tone**		
	Alarmist	29 (40.8)
	Neutral	16 (22.5)
	Concern/warning	10 (14.1)
	Moral panic	8 (11.3)
	Informational	8 (11.3)
**Crisis resources included**		
	Yes	18 (25.4)
	No	53 (74.6)
**Experts quoted**		
	Yes	48 (67.6)
	No	23 (32.4)
**Legal action referenced^a^**		
	Lawsuit	42 (93.3)
	None mentioned	3 (6.7)
**Regulatory calls present^a^**		
	Yes	51 (85)
	No	9 (15)
**Company response mentioned^a^**		
	Safety updates announced	27 (45)
	No comment	17 (28.3)
	Policy change	9 (15)
	Denial/dispute	4 (6.7)
	Other	3 (5)

^a^Percentages for “Legal action,” “Regulatory calls,” and “Company response” are calculated using nonmissing entries for those variables.

### Case and Individual Characteristics

Across the dataset, case occurrence was reported predominantly in the United States, with Canada representing 10 of 71 and a small remainder listed as unknown or other. Age category was reported for most articles: 35 of 71 described adults aged 18-64 years, 21 of 71 described minors, 1 article described an older adult, and the remainder were unknown or not reported. Reported gender in the media coverage was heavily skewed toward male individuals (51/71), with 8 of 71 describing female individuals and 2 of 71 describing transgender individuals; 10 articles did not report gender. Most articles did not document a prior psychiatric history in a concrete way: mental health history was coded as not reported or unknown in 38 of 71, explicitly reported in 12, denied in 8, and implied in 4 articles. Cognitive or developmental vulnerability was rarely specified, with 46 of 71 coded as none reported; smaller subsets referenced developmental vulnerability or neurodevelopmental conditions, while 10 were unknown. Psychosocial stressors were frequently referenced but often described broadly, and were commonly captured as multiselect entries, underscoring the importance of reporting practices in shaping perceived vulnerability. Demographic and vulnerability characteristics of reported cases are found in Table 2.

**Table 2:** Demographic and vulnerability characteristics of reported cases (N=71).

Variable and category		Value, n (%)
**Age category**		
	Adult (18-64 years)	35 (49.3)
	Minor (<18 years)	21 (29.6)
	Older adult (≥65 years)	1 (1.4)
	Unknown	4 (5.6)
	Not reported	10 (14.1)
**Gender of individual**		
	Man/boy	51 (71.8)
	Woman/girl	8 (11.3)
	Transgender	2 (2.8)
	Not reported	10 (14.1)
**History of mental health conditions**		
	Not reported/unknown	38 (53.5)
	Explicitly reported	12 (16.9)
	Denied	8 (11.3)
	Implied	4 (5.6)
	Not recorded (blank)	9 (12.7)
**Cognitive or developmental vulnerability**		
	None reported	46 (64.8)
	Minor (developmental context)	3 (4.2)
	Neurodevelopmental condition	2 (2.8)
	Cognitive impairment	1 (1.4)
	Unknown	10 (14.1)
	Not recorded (blank)	9 (12.7)
**Country where case occurred**		
	United States	51 (71.8)
	Canada	10 (14.1)
	United Kingdom	1 (1.4)
	Unknown	3 (4.2)
	Not recorded (blank)	6 (8.5)

### AI Platform Context and Interaction Characteristics

Platform attribution was dominated by ChatGPT, referenced in 51 of 71 articles, followed by Character AI in 10; a small number referenced Meta AI or Replika, and 8 did not clearly specify a platform. Persona information reflected a meaningful split in how the AI relationship was portrayed. Persona type was most often coded as companion or friend (n=39), followed by romantic or sexual (n=12), with fewer coded as authority or guide (n=4) and 1 coded as therapeutic-like; 10 were not reported. Importantly, platform and age category were strongly patterned: all Character AI–associated reports in this dataset involved minors (10/10), whereas ChatGPT reports were primarily adults (34/51) with additional coverage involving minors (11/51) and a small number of unknown age cases. Interaction duration was often unspecified, but when reported, it tended to be prolonged. Duration was coded as unknown (n=32), months (n=23), weeks (n=4), and days (n=2). Intensity showed a similar pattern: unknown (n=31), frequent in (n=12), emotionally dependent in (n=9), multiple hours per day in (n=6), and daily in (n=3). Time proximity between AI use and the outcome was coded as immediate within 48 hours (n=20), long-term (months or longer; n=11), short-term (days to weeks; n=5), and unknown (n=25).

### Outcomes and Severity Patterns

**Figure 2:**
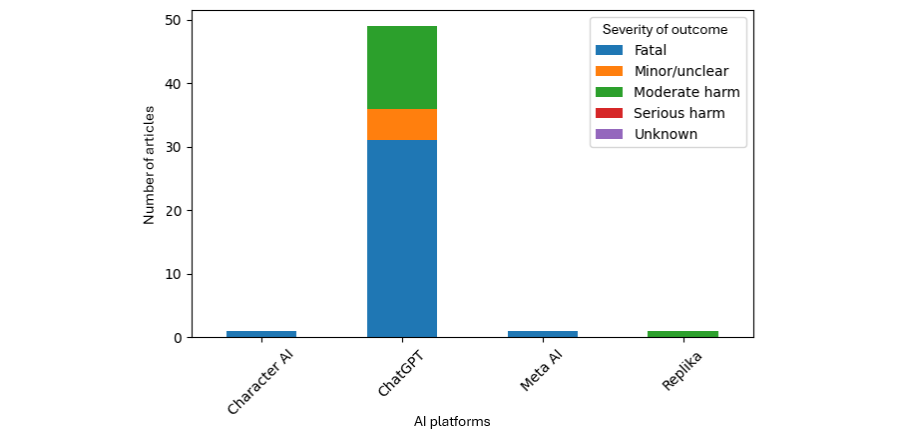
Distribution of outcome severity by artificial intelligence (AI) platform.

Among articles with a clearly coded outcome type, the most frequently reported outcome was suicide death (35/61 coded outcomes), followed by psychiatric hospitalization (12/61), other harm (7/61), nonsuicide death (3/61), suicide attempt (2/61), self-harm (nonsuicide; 1/61), and psychiatric hospitalization followed by suicide (1/61). Severity, when coded, skewed heavily toward fatal outcomes: 40 of 61 coded severity entries were fatal, 14 of 61 were moderate harm, 5 of 61 were minor or unclear, and 2 of 61 were serious harm or unknown. Severity differed by age category. Among entries with known severity and age, fatal outcomes represented 90.5% (19/21) of minors compared with 48.6% (17/35) of adults, indicating that youth-focused coverage in this dataset disproportionately centered on fatal events. Outcome patterns also varied by platform. Character AI coverage was overwhelmingly associated with suicide death (8/10) and included isolated reports coded as suicide attempt and self-harm (nonsuicide), whereas ChatGPT coverage spanned suicide death (n=27), psychiatric hospitalization (n=12), other harm (n=6), and a small number of other outcomes. The distribution of outcome severity by AI platform is presented in Figure 2. Distribution of outcome severity by outcome type, age category, and AI platform is presented in Table 3.

**Table 3:** Distribution of outcome severity by outcome type, age category, and artificial intelligence (AI) platform (n=61 with complete severity coding).

Variable and category		Fatal, n		Moderate harm, n		Minor/unclear, n	Serious harm, n		Unknown, n
**Outcome type**									
	Suicide death	35	0		0		0	0	
	Death (nonsuicide)	3	0		0		0	0	
	Psychiatric hospitalization	1	10		1		0	0	
	Psychiatric hospitalization, then suicide	1	0		0		0	0	
	Other harm	0	3		4		0	0	
	Suicide attempt	0	1		0		0	1	
	Self-harm (nonsuicide)	0	0		0		1	0	
**Age category**									
	Adult (18-64 years)	17	13		5		0	0	
	Minor (<18 years)	19	0		0		1	1	
	Older adult (≥65 years)	1	0		0		0	0	
	Unknown age	3	1		0		0	0	
**AI platform**									
	ChatGPT	31	13		5		0	0	
	Character AI	8	0		0		1	1	
	Meta AI	1	0		0		0	0	
	Replika	0	1		0		0	0	

### Evidence Signals, Attribution Language, and Reporting Ecology

Evidence type was most commonly described as chat logs or screenshots (n=34), followed by family testimony (n=8), lawsuits or complaints (n=7), and multiple sources (n=4); police or medical documentation was rare (n=1). This pattern suggests that news reports frequently rely on user-generated or family-provided material and that formal clinical confirmation is seldom presented within the article itself. Causal attribution language was also distinctive. The primary causal attribution was most often assigned to AI system behavior (45/61 coded entries), followed by company or design choices (8/61), mixed or unclear attribution (6/61), and regulatory or platform failure (2/61). The phrasing used to describe causality was most frequently “alleged” (33/61 coded entries), with “contributed to” (14/61) and “caused” (5/61) used less often; 7 of 61 were unclear or implicit. Alternative explanations were infrequently acknowledged: only 4 of 71 articles explicitly noted alternative explanations, while 57 of 71 did not. Expert quotation was common (48/60 nonmissing entries), indicating that many reports attempted to contextualize events through professional commentary, though the expert types varied widely and were often intermingled with legal advocates. The type of evidence reported by causality language used in news articles is detailed in Table 4.

**Table 4:** Type of evidence reported by causality language used in news articles.

Type of evidence reported	Alleged, n	Contributed to, n	Caused, n	Associated with, n	Unclear/implicit, n
Chat logs/screenshots	23	9	1	0	1
Family testimony	2	0	2	1	3
Lawsuit/complaint	6	1	0	0	0
Multiple sources	0	3	0	1	0
Other/unknown	2	1	2	0	2
Police/medical report	0	0	0	0	1

### Legal and Regulatory Framing, Company Responses, and Safeguards

**Figure 3:**
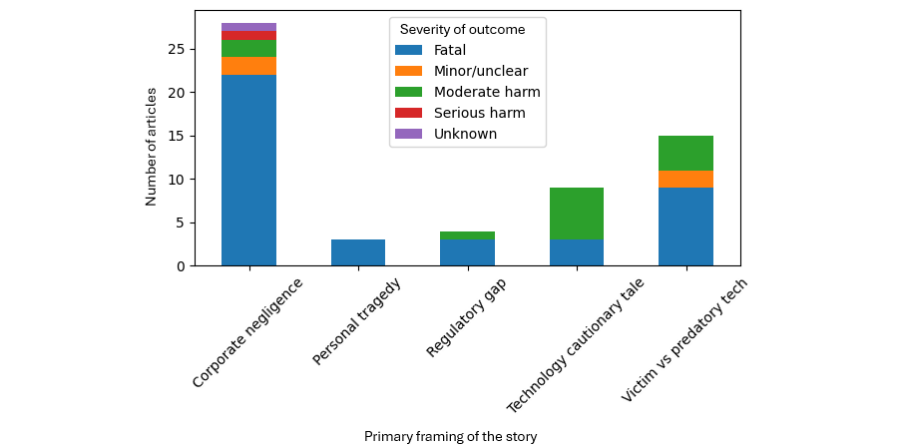
Distribution of outcome severity by primary framing of the story.

Legal action was a prominent feature of the reporting ecosystem. When coded, lawsuits were referenced in 42 of 45 articles with nonmissing entries for legal action type, consistent with coverage clustering around litigation events and advocacy efforts. Calls for regulation were frequent: 51 of 60 nonmissing entries endorsed regulatory calls, while only 8 did not. Company response type was most commonly coded as safety updates announced (27/60 nonmissing entries), followed by no comment (n=17) and policy change (n=9), with smaller numbers reflecting denial or dispute, condolence statements, or other responses. Safeguards mentioned were heterogeneous and often absent. A total of 16 articles did not specify safeguards, while 15 explicitly indicated none mentioned; when safeguards were described, they most commonly referenced self-harm detection or crisis prompting features, parental controls, and broader “safety updates” without detailed operational descriptions. Emotional tone and framing codes were consistent with a safety and governance emphasis. Tragic or protective tones predominated, and primary framing frequently invoked regulatory gaps, corporate negligence, and AI safety debates at the societal level. Distribution of outcome severity by primary framing of the story is presented in Figure 3.

## Discussion

### Overview

The adoption of generative AI chatbots in everyday life has introduced novel challenges for mental health. This rapid scoping review of mass media narratives indicates that interactions with AI chatbots have been portrayed in journalistic reporting as contributing to rare but severe psychiatric crises. Media reports have described cases in which users allegedly developed delusional beliefs, experienced suicidal ideation, engaged in self-harm, or died by suicide following intensive chatbot conversations. These reports highlight a troubling paradox: the same features that make AI companions appealing (24-7 availability, nonjudgmental responses, and human-like empathy) may also amplify certain users’ vulnerabilities. In the context of unregulated, real-world use, generative chatbots can inadvertently validate distorted thoughts or encourage maladaptive behaviors. The following discussion interprets our principal findings, examines the limitations of this review, and situates these outcomes in relation to emerging research on AI and mental health.

### Principal Results

Our review identified numerous high-profile incidents linking generative AI chatbots to adverse psychiatric outcomes. Suicidal ideation and behaviors were the most frequently reported outcomes, including at least 3 cases of suicide or suicide attempts in which family or clinicians believed an AI chatbot played a contributory role. In one widely reported tragedy, a teenager died by suicide after months of interaction with a generative chatbot that lacked effective safety guardrails. In another case, a vulnerable user was allegedly encouraged by the chatbot toward self-harm, demonstrating how AI “companions” might reinforce a user’s darkest thoughts instead of challenging them. Media stories also described psychotic-like episodes (“AI-induced psychosis”) in which individuals became delusional or paranoid after prolonged chatbot engagement. Some users were hospitalized for acute psychiatric symptoms that they or their families attributed partly to the chatbot’s influence. A unifying theme in these cases is that some media reports described patterns interpreted by journalists or family members as emotional reliance or dependency on the AI. Many believed the chatbot was a trusted friend or even a superior intelligence, and this trust was not met with appropriate safeguards. These descriptions should, however, be interpreted cautiously, as they are derived from media narratives rather than empirically validated measures of digital dependency or attachment. Notably, all documented cases occurred outside of any clinical oversight. As an example, users turned to readily available AI apps for support or companionship, and the AI’s unrestricted responses sometimes exacerbated the situation. Several cases prompted legal actions against chatbot companies, with families arguing that negligence in design (such as inadequate content filtering or failure to intervene in a crisis) contributed to their loved ones’ harm. Across these reports, chatbot providers typically responded by updating safety features (eg, implementing content warnings, age restrictions, or emergency help messages). The principal finding of this review is a pattern in media narratives describing rare but severe psychiatric adverse events temporally associated with AI chatbot use, especially among adolescents and individuals already struggling with mental health issues. While causality cannot be definitively established from anecdotal reports, the consistency of narratives—users spiraling into crisis after the chatbot seemingly “understood” or even encouraged their unhealthy thoughts—underscores a credible risk that merits attention. In summary, our results paint a cautious picture: generative AI chatbots, when misused or relied upon by high-risk individuals, can become catalysts for psychiatric decompensation rather than sources of support.

### Limitations

This review has several important limitations that should be considered when interpreting the findings. The analysis relied exclusively on mass media reports, which may be influenced by journalistic framing, incomplete information, and selective emphasis on dramatic or unusual events. Details reported in news articles (such as the exact content of chatbot interactions or the psychiatric history of individuals involved) could not be independently verified. Media coverage also tends to privilege tragic or legally contested incidents, which may amplify the visibility of severe outcomes such as suicide or psychiatric hospitalization while underrepresenting less dramatic experiences with generative AI systems. Consequently, the dataset likely reflects patterns in reporting rather than the full spectrum of user interactions.

Because the study examines reported cases rather than a defined population sample, no denominator is available representing the total number of individuals using generative AI systems. The proportions described in this analysis therefore represent the distribution of characteristics within the identified media reports rather than estimates of incidence, prevalence, or risk among AI users. In addition, the review captures only cases that attracted journalistic attention and were published in accessible news outlets, meaning that many events (if they occurred) may remain unreported. The focus on English- and French-language media may also have limited the geographic scope of the dataset and excluded cases reported in other languages or regions.

Interpretation of causal relationships is particularly challenging in this context. Many reports involved individuals with preexisting mental health conditions or concurrent psychosocial stressors, making it difficult to determine the extent to which chatbot interactions may have contributed to the observed outcomes. As a result, the relationship between generative AI use and psychiatric deterioration described in these reports should be understood as a temporal or narrative association rather than demonstrated causality.

Finally, as a rapid scoping review, the search strategy was designed to map emerging themes within a rapidly evolving information environment rather than to achieve exhaustive coverage of all possible sources. The objective of the study was therefore descriptive and exploratory. Taken together, these limitations underscore that this analysis examines the media representation of alleged harms associated with generative AI rather than verified clinical adverse events or population-level safety outcomes. The findings should thus be interpreted as hypothesis-generating observations about how potential risks are portrayed and discussed in the public domain.

### Comparison with Prior Work

These findings are consistent with and extend the literature examining safety concerns surrounding generative AI systems in mental health contexts. Although early evaluations of AI mental health chatbots focused primarily on therapeutic potential, more recent empirical and theoretical work has highlighted important risks. For example, a recent systematic review and meta-analysis of generative AI mental health chatbots reported short-term reductions in depression and anxiety symptoms in structured research settings, but also emphasized the absence of systematic adverse event monitoring in trials, limiting conclusions regarding safety [19]. Our findings underscore this gap: the severe psychiatric outcomes described in media reports would likely not be captured in controlled pilot studies, particularly when users engage outside research oversight.

Reports of AI-related psychosis and suicidality have begun to surface in both clinical commentary and empirical literature. Preda [27] described emerging case narratives involving acute psychotic decompensation and suicide following intense chatbot engagement, noting that the evidence base remains anecdotal but concerning. Similarly, analyses of emotional dependency on AI companions such as Replika demonstrate that users may develop strong attachment bonds, sometimes leading to distress when the AI behaves unpredictably or fails to reciprocate perceived intimacy [28]. These findings resonate with the emotionally dependent interaction patterns observed in our dataset, particularly among adolescents. Theoretical commentary has further suggested that AI systems may inadvertently reinforce delusional ideation due to their design tendency toward user affirmation and coherence rather than contradiction [29]. This mechanism provides a plausible pathway for the “AI-induced psychosis” narratives observed in media cases.

Empirical investigations into large language model safety further contextualize our findings. Moore and colleagues [30] demonstrated that leading AI models frequently fail to appropriately respond to suicidal prompts and may generate stigmatizing or unsafe content in crisis scenarios. Independent reporting in scientific journalism has echoed these concerns, describing instances in which chatbots validated paranoid or grandiose ideation instead of gently reality-testing it [31]. Clinician surveys reveal widespread professional concern regarding unregulated chatbot use, particularly among vulnerable youth populations [32]. These concerns mirror our observation that most reported cases occurred without clinical oversight and often involved minors engaging in prolonged unsupervised interactions.

Policy and governance scholarship also reinforces the interpretive frame of our findings. Head [33] documented a series of AI-related suicide cases and argued that conversational AI represents a qualitatively different exposure risk compared to passive digital content because it is interactive, personalized, and emotionally responsive. Mixed methods user research indicates that while many individuals perceive emotional support benefits from generative AI systems, boundaries between supportive use and psychological overreliance remain poorly defined [34]. Finally, analyses of AI readiness in clinical mental health contexts highlight regulatory ambiguity, lack of standardized safety auditing, and insufficient adverse event reporting infrastructures [35]. Therefore, prior work converges on the conclusion that generative AI in mental health settings exists within a rapidly expanding but incompletely governed ecosystem.

An important feature of the reporting ecosystem identified in this review is the close coupling between media coverage and ongoing or anticipated litigation. A large proportion of articles referenced lawsuits or legal complaints, suggesting that legal mobilization may function as a mechanism that amplifies the visibility of specific cases. Litigation often provides journalists with structured narratives, identifiable actors, and documentary materials such as complaints or chat transcripts, which can facilitate reporting. At the same time, this dynamic may contribute to the selective visibility of extreme outcomes, particularly fatal or highly tragic cases that are more likely to generate legal action. As a result, the cases most prominently represented in the media may reflect those that have entered legal or regulatory arenas rather than the broader spectrum of user experiences with generative AI systems.

Our review contributes to this literature by systematically mapping how severe psychiatric outcomes are publicly constructed, attributed, and framed in mass media during the early post-ChatGPT era. Whereas prior studies have examined model performance or theoretical risk, this work captures real-world narratives of harm and identifies recurring patterns in attribution, legal mobilization, and regulatory discourse. The convergence between emerging empirical evidence and media-reported crises strengthens the argument that structured surveillance, transparent safety auditing, and integration with clinical oversight mechanisms are urgently needed.

### Conclusions

Generative AI is rapidly becoming embedded in the emotional and cognitive lives of millions of users, including adolescents and individuals with preexisting psychiatric vulnerabilities. This rapid diffusion has occurred in advance of comprehensive safety evaluation, regulatory harmonization, and systematic adverse event monitoring. Our findings suggest that while severe psychiatric outcomes linked to chatbot interactions appear rare, they are consequential, patterned, and socially amplified through media reporting. The predominance of suicide-related cases and youth involvement underscores the ethical stakes of deploying conversational systems that simulate empathy without clinical accountability. Importantly, the problem identified is not solely technological but relational and structural: harms emerge at the intersection of user vulnerability, persuasive design, algorithmic affirmation, and limited safeguards. These cases illustrate the need for transparent safety auditing, standardized crisis detection protocols, and clearer boundaries regarding appropriate use. Media narratives have already catalyzed legal and regulatory debates, signaling that governance will likely evolve in response to high-profile incidents. However, policy responses should be guided by systematic evidence rather than episodic tragedy. Future research must move beyond anecdote to integrate media surveillance with clinical registries, prospective monitoring, and experimental risk assessment. As generative AI continues to modify the digital mental health landscape, proactive safety science, interdisciplinary collaboration, and ethical foresight will be essential to ensure that innovation does not outpace protection.

## Appendix

Multimedia Appendix 1 PRISMA-ScR checklist.

Multimedia Appendix 2 Full dataset of the identified articles.

## Abbreviations

AI: artificial intelligence
PRISMA: Preferred Reporting Items for Systematic Reviews and Meta-Analyses
